# Miniaturized Biomedical Sensors for Enumeration of Extracellular Vesicles

**DOI:** 10.3390/ijms19082213

**Published:** 2018-07-29

**Authors:** Anil Kumar Pulikkathodi, Indu Sarangadharan, Chiao-Yun Lo, Po-Hsuan Chen, Chih-Chen Chen, Yu-Lin Wang

**Affiliations:** 1 Institute of NanoEngineering and MicroSystems, National Tsing Hua University, Hsinchu 300, Taiwan; anilpnarayan@gmail.com (A.K.P.); indu.4391@gmail.com (I.S.); st2525dog@gmail.com (C.-Y.L.); sam76227@gmail.com (P.-H.C.); 2 Department of Power Mechanical Engineering, National Tsing Hua University, Hsinchu 300, Taiwan

**Keywords:** extracellular vesicles, GaN HEMT biosensor, in-vitro diagnostics, high field modulated FET

## Abstract

In this research, we have realized a rapid extracellular vesicle (EV) quantification methodology using a high field modulated AlGaN/GaN high electron mobility (HEMT) biosensor. The unique sensing structure facilitated the detection of the sub-cellular components in physiological salt environment without requiring extensive sample pre-treatments. The high field operation of GaN HEMT biosensor provides high sensitivity and wide dynamic range of detection of EVs (10^7^–10^10^ EVs/mL). An antibody specific to the known surface marker on the EV was used to capture them for quantification using an HEMT biosensor. Fluorescence microscopy images confirm the successful capture of EVs from the test solution. The present method can detect EVs in high ionic strength solution, with a short sample incubation period of 5 min, and does not require labels or additional reagents or wash/block steps. This methodology has the potential to be used in clinical applications for rapid EV quantification from blood or serum for the development of diagnostic and prognostic tools.

## 1. Introduction

Extracellular vesicles (EVs) are membrane-bound sub-cellular components that are released by cells [1]. They are used for cellular communication and can carry biomolecular cargo including proteins, nucleotides and lipids [1,2,3]. Due to their roles in cellular communication, EVs have attracted great interest in assessing their potential as a diagnostic and prognostic biomarker. EVs take part in several patho-physiological processes such as homeostasis, immune response and their modulatory effects, and disease progression such as cancer [4,5]. EVs can be classified according to biogenesis into three main categories: exosomes, microvesicles and apoptotic bodies, with exosomes being the most widely researched. EVs provide functional and structural protection to their cargo and possess longer half-life in blood, which enhances their diagnostic potential [6]. Quantifying the amount of EVs and analyzing the type and contents may provide deeper understanding of the aberrant process leading to diseases. Peripheral blood carries abundant EVs which circulate throughout the body and blood-based assays can be used to capture, quantify and analyze EVs.

Presently, conventional techniques are being developed and optimized for the quantification of EVs. Nanoparticle tracking analysis (NTA) is the most commonly used EV quantification method with a dynamic range of 10^7^–10^10^ particles/mL [7]. Flow cytometry has also been used in the phenotyping and enumeration of EVs [8]. Apart from traditional methodologies, newer techniques such as scanning ion occlusion sensing are being developed, which provide greater scope for rapid EV quantification [9]. However, these methodologies are complex and sophisticated requiring bench-top instruments, in addition, they lack selectivity and require extensive pre-processing such as filtering and purification, as well as additional reagents and labels. This limits the clinical applications of rapid EV-based diagnostics. 

Miniaturization technologies have led to the development of compact and automated diagnostic platforms such as microfluidics-based sub-cellular diagnostics [10]. Electronic sensors based on semiconductor devices such as field effect transistor (FET)-based biosensors garner importance in miniaturized systems due to their high sensitivity, short response times, easy signal read out capabilities and label-free detection method [11,12]. However, clinical applications of FET biosensors were limited due to the charge screening effect in high ionic strength solutions such as physiological fluids which demand extensive sample pre-treatments [13,14,15]. In our previous works, we developed a new FET bio-sensing methodology using AlGaN/GaN high electron mobility transistor (HEMT) for protein detection in physiological salt concentration [16,17,18]. We were able to directly detect target proteins in clinical sera, even with the presence of large quantities of non-specific proteins in the plasma proteome.

In the present research, we have developed AlGaN/GaN HEMT biosensors for the detection and enumeration of EVs in a physiological salt environment, without requiring dilution or fractionation. We have focused on quantifying the exosomes extracted from human embryonic kidney (HEK-293T) cells. GaN HEMT offers biocompatibility in addition to performing with superior electrical characteristics. Since the HEMT biosensor is operated as an electrical double layer (EDL) gated FET sensor, it exhibits high sensitivity and a wide dynamic range of detection (10^7^–10^10^ EVs/mL). The capture of EVs using anti-CD63 is confirmed using fluorescence microscopy. This technology can perform rapid EV quantification in physiological fluids without requiring extra reagents or labeling and has demonstrated its potential to be used as a point-of-care in vitro diagnostic device.

## 2. Results and Discussion

### 2.1. Structure of GaN HEMT Biosensor

**Figure 1:**
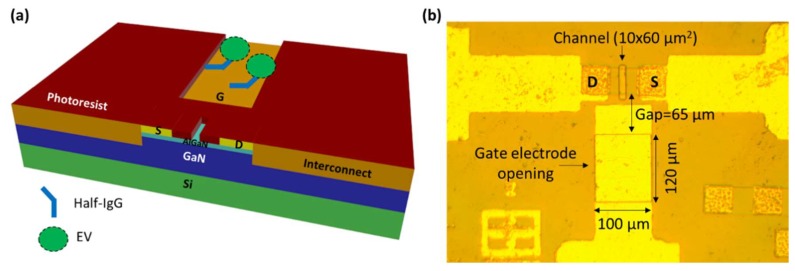
(**a**) Schematic illustration of AlGaN/GaN HEMT biosensor for EV quantification. (**b**) Top view image of GaN HEMT biosensor showing the sensing region comprised of gate electrode opening and transistor channel opening separated by 65 µm gap.

The structural information of the biosensor is depicted in Figure 1. The sensing region comprises of the openings on the gate electrode and the transistor channel, separated by a very short gap of 65 µm. The separated gate electrode is in fact the reference electrode used in conventional FET biosensors, but re-purposed to provide high field modulation to FET sensing. The schematic illustration of GaN HEMT biosensor is depicted in Figure 1a, which shows the gate electrode functionalized with the receptor, in this case, antibody, which targets the exosomes from the test solution. The real top view image of the GaN HEMT biosensor device is shown in Figure 1b. The opening on the channel region is 10 × 60 µm^2^ in an area separated by 65 µm from the gate electrode opening, which is 100 × 120 µm^2^ in area. The test solution containing the target exosomes is dropped on the sensor surface such that it forms the dielectric between the gate electrode and channel openings. Assuming the HEMT channel has negligible resistance, the sensing structure can be regarded as two metal electrodes of asymmetric sizes connected by an electrolyte which is the test solution. 

The working principle of our GaN HEMT biosensor is detailed in our previous works [16,17,18]. Briefly, when the test solutions containing high salt concentration (physiological salt concentration is ~150 mM) are dropped on the sensor surface and a pulsed gate voltage is applied, the electrical double layer (EDL) on the solid/liquid interfaces (the interfaces of solution and gate electrode and channel) is re-distributed, generating a solution capacitance.

The applied potential drops across the test solution and the transistor dielectric, which can be expressed as:

(1) $$V_{g}=V_{s}+V_{d}$$

where *V_g_*, *V_s_* and *V_d_* are the applied gate potential, potential drop across solution and transistor dielectric (AlGaN), respectively.

Impedance in this system can be equated as:

(2) $$Z=\frac{1}{j\omega C}$$

The sensing structure yields a purely capacitive response, which is verified by measuring the gate electrode leakage current. The total capacitance in this system can be written as:

(3) $$\frac{1}{C_{total}}=\frac{1}{C_{s}}+\frac{1}{C_{d}}$$

where *C_s_* and *C_d_* are the solution and transistor dielectric capacitances, respectively. When the overall impedance is high, the voltage drop is also large. The ratio of impedances in the solution and dielectric determines the overall potential drop across the dielectric. The numerator represents the impedance in the transistor dielectric and denominator represents the summation of impedances in the solution and dielectric. Therefore, combining Equations (1)–(3) we can express the potential drop across the dielectric in terms of applied gate voltage and solution capacitance as:

(4) $$V_{d}=\frac{\frac{1}{j\omega C_{d}}}{\frac{1}{j\omega C_{d}}+\frac{1}{j\omega C_{s}}}V_{g}=\frac{C_{s}}{C_{s}+C_{d}}V_{g}$$

The solution capacitance modulates the potential drop across the transistor dielectric (i.e., AlGaN) and hence the drain current of HEMT. Therefore, at higher ionic strength, higher drain current signal is observed, owing to larger solution capacitance [16]. The solution capacitance can also change due to surface functionalization and receptor-ligand binding, whereby changing the drain current signal. In this way, we are able to monitor the biological interactions on the sensor surface as the electrical response from the GaN HEMT sensor, which is explained in the following sections. Throughout this study, we have used current gain as the sensor signal instead of absolute drain current. Current gain analogous to the transconductance gm of FET is defined as the difference of transistor drain current before and after applying gate voltage *V_g_*. Current gain or simply referred to here as “gain” is a relatively more stable index for sensor measurement compared to the absolute drain current of the transistor as the latter may be prone to variations from external and thermal noise.

### 2.2. Surface Functionalization and Characterization

**Figure 2:**
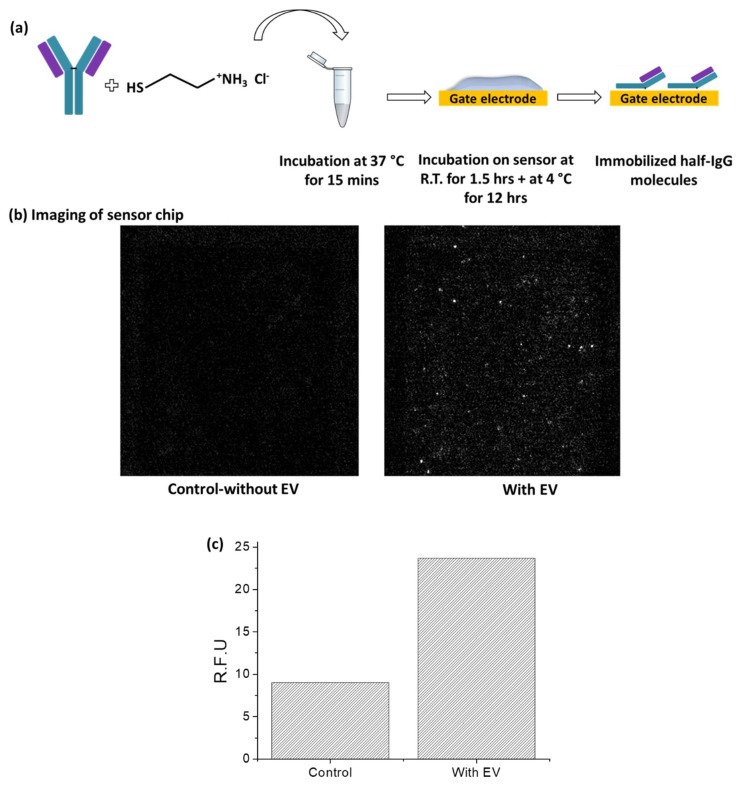
(**a**) Schematic illustration of surface functionalization process. (**b**) Imaging functionalized sensor chip with and without EVs. Left hand side image of the gate electrode represents control i.e., functionalized sensor without EVs. Right hand side image of the gate electrode shows the functionalized sensor with captured EVs. (**c**) Relative fluorescence intensity of functionalized sensor chip with and without EVs.

We have targeted known exosome surface markers to capture them from the test solution using corresponding antibodies. Tetraspanins such as CD63 used for exosome formation and secretion have been commonly used for exosome purification and enrichment [19]. We therefore used anti-CD63 to capture the EVs from the test solution. The surface functionalization involves immobilization of anti-CD63 on the gate electrode opening using the native thiol groups in the IgG molecule to covalently bind with Au surface. The protocol is schematically illustrated in Figure 2a. Since the antibodies are immobilized onto the surface via their hinge regions, the binding sites remain in the proper orientation, suitable for unobstructed binding with the ligand. We have characterized the surface functionalization in two methods: using optical imaging and electrical measurements of the HEMT sensor. EVs used in our study are fluorescent and express fusion proteins on their exosomal membranes that fluoresce under blue light excitation [20]. Therefore, antibody immobilization can be confirmed by imaging the gold gate electrode surface before and after incubation with EVs. After immobilizing anti-CD63 on the gold surface, EVs were allowed to be captured by the antibodies, by incubation in room temperature for 1 h. Unbound EVs were washed away and fluorescence microscope was used to record images of EVs captured by anti-CD63. Figure 2b shows the optical image of EVs captured by anti-CD63 on gold gate electrode opening on the HEMT sensor chip. The control image in Figure 2b was recorded prior to incubation with EVs (sensor functionalized with anti-CD63). Figure 2c shows the relative fluorescence intensity of the functionalized sensor with and without EVs. The fluorescence intensity when EVs are captured is higher than the control. The capture of EVs on the gold electrode confirms that anti-CD63 is immobilized on the sensor and can bind with the CD63 expressed on the exosomal membranes. 

**Figure 3:**
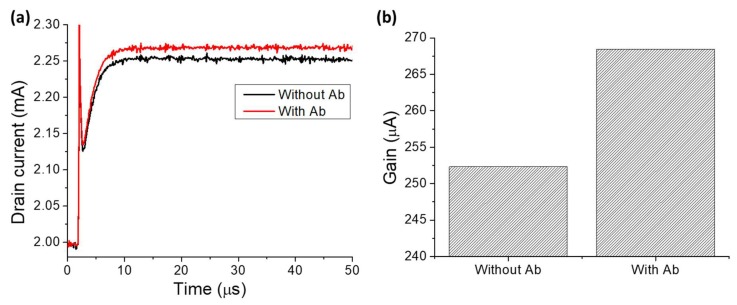
(**a**) Drain current versus time before and after surface functionalization. (**b**) Gain response before and after surface functionalization.

The surface functionalization can also be monitored electrically. The interface of the solution and gate electrode changes as IgG molecules are bound to the electrode surface. This follows that when the electric field is applied across the test solution, EDL on the gate electrode interface is modified, which leads to a corresponding charge re-distribution in the EDL on the solution-transistor dielectric region. This leads to a change in solution capacitance and hence the drain current response of the HEMT sensor. Therefore, by monitoring the drain current before and after surface functionalization, as shown in Figure 3a, we can indeed characterize the antibody immobilization procedure. Figure 3b shows the change in current gain of HEMT biosensor upon surface functionalization, which is the differential drain current signal that is used as the sensor index of measurement throughout this study.

### 2.3. Detection and Enumeration of EVs

**Figure 4:**
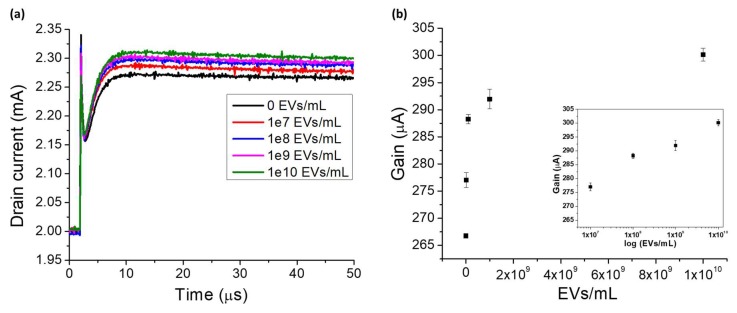
(**a**) Drain current versus time for different EV concentrations. (**b**) Sensor response curve for EV enumeration. Inset shows response in log scale.

The sensing characteristics of GaN HEMT biosensor are demonstrated by the quantitative measurement of different concentrations of EVs on the sensor surface. The sensor response measured after surface functionalization with anti-CD63 is used as the baseline for further measurements with EVs. EV concentrations in the range of 10^7^–10^10^ EVs/mL are used in this study. Figure 4a shows the drain current signal of the HEMT sensor for different EV concentrations. The drain current increases as the number of EVs increases. This is because when the anti-CD63 binds with the CD63 expressed on the exosomal membrane and when gate pulse voltage is applied, the EDLs at the gate electrode and transistor dielectric interfaces undergo charge re-distribution, which changes the solution capacitance. As seen from Equation (4), at fixed gate voltage when solution capacitance is varied, the potential drop across the transistor dielectric is varied and as a result the drain current of HEMT changes. The channel of HEMT is composed of 2-dimensional electron gas (2DEG) which is highly sensitive to changes in the gate potential. As the number of captured EVs on the gate electrode increases, the potential drop across the solution decreases, causing more potential to drop across the transistor dielectric. This leads to larger changes in the drain current of HEMT. The change in drain current is thus proportional to the number of EVs captured at the sensing region. We use current gain as the sensor measurement index to avoid the inconsistencies in absolute drain current. Figure 4b shows the calibration curve of the HEMT biosensor, with current gain versus the number of EVs captured on the sensor. The inset demonstrates the sensor response curve in logarithmic scale, to better represent the linearity of sensor response over the wide dynamic range of detection of EVs. The results in Figure 4 demonstrate that our GaN HEMT biosensor can be used to enumerate EVs in the range of 10^7^–10^10^ EVs/mL, which includes two orders lower and one order higher than the normal EV concentration in humans which is 10^9^ EV/mL [21,22]. Thus, our electronic microsensor can detect and enumerate EVs with very high sensitivity in physiological salt environment, without requiring additional sample pre-processing or extra reagents/labels. The dynamic range of detection exhibited by our GaN HEMT sensor is comparable to the gold standard EV detection methodology of nanoparticle tracking analysis (10^7^–10^10^ EVs/mL). Enumeration of EVs has the potential for risk assessment of cardiovascular diseases like coronary artery disease and early diagnosis of several types of cancers such as ovarian, lung and gastric cancer [23,24,25,26,27]. Therefore, our electronic microsensor has the potential to improve point of care diagnostics for diseases that cause the largest mortality worldwide.

The high field operation of the HEMT sensor is the reason for higher sensitivity in physiological salt concentration. As seen from Equations (1) and (4), higher *V_g_* or higher solution capacitance can lead to a larger potential drop across the transistor dielectric. In our sensor operation, we provide 2 V as gate voltage which is sufficiently large to acquire the desired sensitivity but not affect the activity of biomolecules or cause redox currents. More importantly, the gap between the gate electrode and the transistor channel is very short (65 µm), which enhances the electric field applied across the test solution. Using this structure, we can operate at larger fields compared to the traditional FET sensors and obtain higher values of solution capacitance. The traditional FET biosensors are not supplied with a high enough external electric field to modulate the channel conductivity and merely rely on the potential changes brought upon by receptor-ligand binding to gate the channel current. Even in cases that use a biased reference electrode (very small bias), the distance between the electrode and the transistor active area is too large and therefore the external bias does not modulate the channel current. Due to severe charge screening effect in physiological salt solution, the potential changes induced by receptor-ligand binding cannot modulate the channel conductivity, which is the reason for demanding extensive sample pre-treatments while using traditional FET biosensors [12,13,14,15]. But using our sensing structure, we have been able to overcome the charge screening effect and directly detect biological targets and ionic species in high salt concentration environments [16,17,18,28]. In our sensor design, we can enhance the sensitivity of our HEMT biosensor to EVs in physiological salt environment. This is an important feature of our HEMT biosensor that holds great potential in clinical applications particularly in IVD and point-of-care testing.

### 2.4. Sensor Regeneration

**Figure 5:**
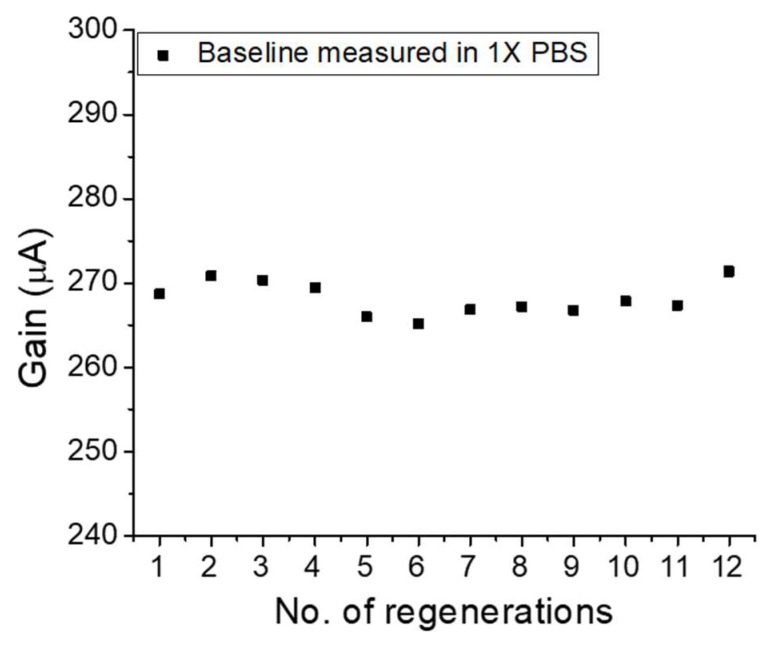
Gain versus number of regenerations after EV detection of HEMT biosensor.

After each sample of EV is measured, the sensor is thoroughly washed in protein elution buffer and 1× PBS to remove captured EVs and non-specific binding, if any. The washing procedure is detailed in Methods Section 2.4. We use a gentle elution buffer with near neutral pH, containing very high salt concentration. The high salt in the buffer is intended to break the electrostatic interactions between the receptor and ligand, thereby eluting them. 1× PBS is used to further wash away the elution buffer and serves as the test environment for further measurements. The electrical baseline of the sensor is measured to confirm whether it has returned to the original reference value, which corresponds to that of the anti-CD63 level. The electrical baseline values between each sample measurement is shown in Figure 5 in terms of gain responses of the HEMT biosensor. The current gain returns to the initial reference value, with each elution procedure, thereby ensuring that the change in gain observed in the sensor calibration curve is indeed resulting from the capture of EVs and not due to non-specific binding. In this study where the four different target EV concentrations have been tested for three different times, the sensor has been regenerated at least 12 times. The results in Figure 5 demonstrate the sensor regeneration capabilities which allow our sensor to be re-used for multiple EV detection and enumeration cycles.

## 3. Materials and Methods

### 3.1. AlGaN/GaN HEMT Fabrication

The epi-wafer consisted of 260 Å AlGaN and 10 Å GaN cap layer deposited using a Metal-organic Chemical Vapor Deposition (MOCVD) system on 3 µm GaN buffer layer on top of 1 mm silicon substrate [29,30]. Active regions of transistor were formed using inductively coupled plasma (ICP) etching performed using BCl_3_/Cl_2_ gas mixture, creating mesa isolations on the epi-wafer. Ohmic contacts were then deposited using an electron beam (E-Beam) evaporation method. The composition of ohmic contacts was Ti/Al/Ni/Au (200/800/400/1000 Å). Following this, rapid thermal annealing (RTA) was carried out at 850 °C in N_2_ environment. To fabricate metal interconnects and the separated gate electrode, E-Beam was used to deposit Ti/Au (200/2000 Å). The miniaturized HEMT chip was packaged using a simple and robust packaging method previously developed [31]. Briefly, a PDMS mold was prepared from a PMMA master and an HEMT chip was aligned and placed on the PDMS mold; the chip pressed on to the mold. Then thermo-curable epoxy resin was poured over the mold and cured at 125 °C and 165 °C for 1 and 1.5 h respectively. Post curing, the HEMT embedded epoxy was peeled off from the mold and metal interconnects were laid using the E-Beam evaporator. The entire chip was passivated using photoresist and sensing regions were opened using photolithography.

### 3.2. Sensor Surface Functionalization

Monoclonal antibody (mAb) to CD63 on the EV membrane was used to capture EVs from the test solution. The anti-CD63 was obtained from System Biosciences. The antibody immobilization on the gate electrode was carried out as a two-step process. A mild reducing agent such as 2-mercaptoethylamine (2-MEA) was used to selectively reduce the disulfide bonds in the hinge region of IgG, resulting in free thiols to bind with gold gate electrode. The anti-CD63 and reducing agent mixture (in a molar ratio of 1:10,000) were incubated in a reaction tube at 37 °C for 15 min and dropped on the gold gate electrode and further incubated for 1.5 h at room temperature. The sensor chip was then incubated at 4 °C for 12 h following which the unbound antibodies were thoroughly washed away in 1X PBS.

### 3.3. Cell Culture and EV Production and Characterization

The cell model from which EVs were derived is a HEK-293T cell line stably expressing palmitoylated green fluorescent protein (GFP), a generous gift from C. P. Lai (Institute of Atomic and Molecular Sciences, Academia Sinica, Taipei, Taiwan). Cells were cultured in high-glucose Dulbecco’s modified Eagle’s medium (DMEM, Corning Cellgro, Manassas, VA, USA) supplemented with 10% fetal bovine serum (FBS, Sigma, St. Louis, MO, USA), 100 U/mL penicillin, and 100 μg/mL streptomycin (Invitrogen, Carlsbad, CA, USA) in a humidified incubator with 5% CO_2_ at 37 °C. To isolate EVs, conditioned medium was collected from ~50% confluent cells incubated with culture medium without FBS for 48 h, followed by sequential centrifugation of the supernatant at 1000× *g* for 10 min and 3000× *g* for 20 min at 8 °C and filtration at 3000× *g* for 1 h and dilution with 2 mL PBS and a subsequent filtration at 3000× *g* for 1 h at 8 °C using an Amicon Ultra-15 10 k centrifugal filter (Millipore, Billerica, MA, USA). Concentrate was collected.

**Figure 6:**
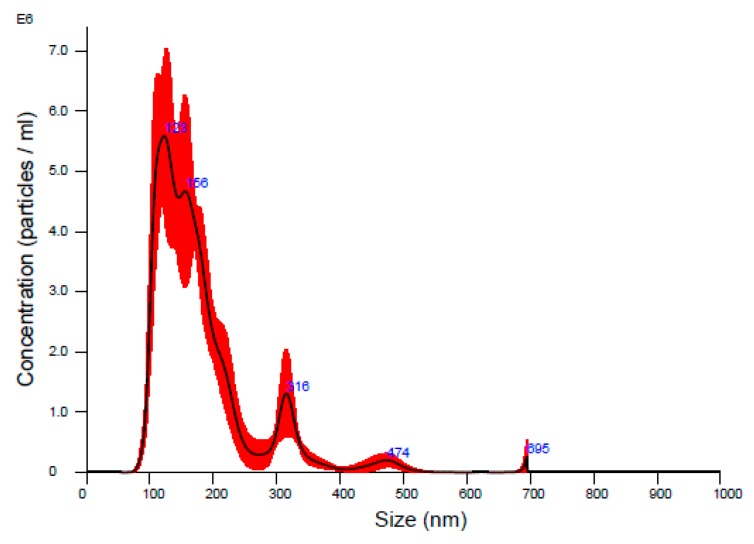
Size distribution from NTA measurements of an 80-fold diluted EV stock solution. The mean and mode diameter are 179 nm and 123 nm, respectively. The concentration is 6.0 × 10^8^ particles/mL.

Concentrated EV samples were diluted 80-fold with 0.22 μm-membrane-filtered PBS and subjected to an NS300 nanoparticle analyzer (NanoSight, Salisbury, UK) to determine the concentration and size distribution using NTA software version 3.2. Stock EV concentration is 4.8 × 10^10^ EVs/mL as assessed by using NTA as shown in Figure 6. 

### 3.4. Sensor Regeneration

After measuring the test samples containing EVs, the sensor chip was thoroughly washed in a mild protein elution buffer (Pierce gentle ag/ab elution buffer, pH 6.6) followed by washing in 1× PBS. The sensor chip was soaked in protein elution buffer for 10 min, followed by washing in 3 mL each of protein elution buffer and 1× PBS. This process was repeated for a period of 30 min. After the final washing step, traces of elution buffer were completely removed by washing the sensor chip in 1× PBS thrice. After this, the electrical baseline of the sensor was measured to confirm sensor regeneration. 

### 3.5. Sensor Measurements

The sensor was characterized by electrical measurements conducted using Agilent B1530/B1500A semiconductor parameter analyzer. The GaN HEMT device was not operated solely under DC bias conditions to avoid excessive heat generation problems. The transistor was applied with a constant source drain bias of 2.5 V and a short duration pulsed gate voltage of 2 V. The gate voltage was applied to the gate electrode and constant DC bias to the drain terminal of HEMT was turned off after pulse application. Initially, drain bias was turned on during which time the gate voltage was held at 0 V for 2 µs. Then the gate pulse was turned on and held at 2 V for 50 µs, after which, the gate and drain biases were turned off. The change in transistor drain current before and after applying the pulsed gate voltage, is defined as current gain of the sensor.

## 4. Conclusions

In this research, we have developed an electronic microsensor based on AlGaN/GaN HEMT for the detection and enumeration of EVs in physiological salt environment. The rapid EV sensing assay does not require any sample pre-treatment procedures such as dilution or filtering. The high field modulated FET operation facilitates sub-cellular components detection in high ionic strength, overcoming the charge screening problem of FET biosensors. Since our HEMT biosensor is EDL gated under high field operation, it offers very high sensitivity, with a wide dynamic range of detection (10^7^–10^10^ EVs/mL), which is better than the conventional EV measurement techniques. Since EVs are directly detected in physiological conditions, our biosensor has the potential to be used in clinical applications that quantify the EVs present in blood for disease diagnosis and characterization [21]. This methodology does not require additional reagents/labels or tedious blocking/washing processes. With high sensitivity, extremely low sample volume requirement (~5 µL), speedy detection (5 min) and minimal assay protocols, our sensor displays great potential to be employed in clinical EV investigations and in the analysis and study of specific EV biology.

## References

[1] Van Niel G., D’Angelo G., Raposo G. (2018). Shedding light on the cell biology of extracellular vesicles. Nat. Rev. Mol. Cell Biol..

[2] Colombo M., Raposo G., Théry C. (2014). Biogenesis, Secretion, and Intercellular Interactions of Exosomes and Other Extracellular Vesicles. Ann. Rev. Cell Dev. Biol..

[3] Lo Cicero A., Stahl P.D., Raposo G. (2015). Extracellular vesicles shuffling intercellular messages: For good or for bad. Curr. Opin. Cell Biol..

[4] Marine M., Liliana P., Maëva V., Ramaroson A., Gilles S., Carmen M.M. (2018). Extracellular Vesicles: Mechanisms in Human Health and Disease. Antioxid. Redox Signal..

[5] Bebelman M.P., Smit M.J., Pegtel D.M., Baglio S.R. (2018). Biogenesis and function of extracellular vesicles in cancer. Pharmacol. Ther..

[6] Johnstone R.M., Adam M., Hammond J.R., Orr L., Turbide C. (1987). Vesicle formation during reticulocyte maturation. Association of plasma membrane activities with released vesicles (exosomes). J. Biol. Chem..

[7] Gardiner C., Ferreira Y.J., Dragovic R.A., Redman C.W.G., Sargent I.L. (2013). Extracellular vesicle sizing and enumeration by nanoparticle tracking analysis. J. Extracell. Vesicles.

[8] Mobarrez F., Antovic J., Egberg N., Hansson M., Jörneskog G., Hultenby K., Wallén H. (2010). A multicolor flow cytometric assay for measurement of platelet-derived microparticles. Thromb. Res..

[9] Garza-Licudine E., Deo D., Yu S., Uz-Zaman A., Dunbar W.B. Portable nanoparticle quantization using a resizable nanopore instrument—The IZON qNano^TM^. Proceedings of the 2010 Annual International Conference of the IEEE Engineering in Medicine and Biology.

[10] Chen C., Lin B.-R., Hsu M.-Y., Cheng C.-M. (2015). Paper-based Devices for Isolation and Characterization of Extracellular Vesicles. J. Vis. Exp..

[11] Chen K.-I., Li B.-R., Chen Y.-T. (2011). Silicon nanowire field-effect transistor-based biosensors for biomedical diagnosis and cellular recording investigation. Nano Today.

[12] Arya S.K., Wong C.C., Jeon Y.J., Bansal T., Park M.K. (2015). Advances in Complementary-Metal–Oxide–Semiconductor-Based Integrated Biosensor Arrays. Chem. Rev..

[13] Elnathan R., Kwiat M., Pevzner A., Engel Y., Burstein L., Khatchtourints A., Lichtenstein A., Kantaev R., Patolsky F. (2012). Biorecognition Layer Engineering: Overcoming Screening Limitations of Nanowire-Based FET Devices. Nano Lett..

[14] Stern E., Wagner R., Sigworth F.J., Breaker R., Fahmy T.M., Reed M.A. (2007). Importance of the Debye Screening Length on Nanowire Field Effect Transistor Sensors. Nano Lett..

[15] Stern E., Vacic A., Rajan N.K., Criscione J.M., Park J., Ilic B.R., Mooney D.J., Reed M.A., Fahmy T.M. (2010). Label-free biomarker detection from whole blood. Nat. Nano.

[16] Chu C.-H., Sarangadharan I., Regmi A., Chen Y.-W., Hsu C.-P., Chang W.-H., Lee G.-Y., Chyi J.-I., Chen C.-C., Shiesh S.-C. (2017). Beyond the Debye length in high ionic strength solution: Direct protein detection with field-effect transistors (FETs) in human serum. Sci. Rep..

[17] Sarangadharan I., Regmi A., Chen Y.-W., Hsu C.-P., Chen P.-C., Chang W.-H., Lee G.-Y., Chyi J.-I., Shiesh S.-C., Lee G.-B. (2018). High sensitivity cardiac troponin I detection in physiological environment using AlGaN/GaN High Electron Mobility Transistor (HEMT) Biosensors. Biosens. Bioelectr..

[18] Chen P.-C., Chen Y.-W., Sarangadharan I., Hsu C.-P., Chen C.-C., Shiesh S.-C., Lee G.-B., Wang Y.-L. (2017). Editors’ Choice—Field-Effect Transistor-Based Biosensors and a Portable Device for Personal Healthcare. ECS J. Solid State Sci. Technol..

[19] Andreu Z., Yáñez-Mó M. (2014). Tetraspanins in Extracellular Vesicle Formation and Function. Front. Immunol..

[20] Reátegui E., van der Vos K.E., Lai C.P., Zeinali M., Atai N.A., Aldikacti B., Floyd F.P., Khankhel H.A., Thapar V., Hochberg F.H. (2018). Engineered nanointerfaces for microfluidic isolation and molecular profiling of tumor-specific extracellular vesicles. Nat. Commun..

[21] Revenfeld A.L.S., Bæk R., Nielsen M.H., Stensballe A., Varming K., Jørgensen M. (2014). Diagnostic and Prognostic Potential of Extracellular Vesicles in Peripheral Blood. Clin. Ther..

[22] Vlassov A.V., Magdaleno S., Setterquist R., Conrad R. (2012). Exosomes: Current knowledge of their composition, biological functions, and diagnostic and therapeutic potentials. Biochim. Biophys. Acta Gen. Subj..

[23] Jansen F., Nickenig G., Werner N. (2017). Extracellular Vesicles in Cardiovascular Disease. Circ. Res..

[24] Szajnik M., Derbis M., Lach M., Patalas P., Michalak M., Drzewiecka H., Szpurek D., Nowakowski A., Spaczynski M., Baranowski W. (2013). Exosomes in Plasma of Patients with Ovarian Carcinoma: Potential Biomarkers of Tumor Progression and Response to Therapy. Gynecol. Obstet..

[25] Baran J., Baj-Krzyworzeka M., Weglarczyk K., Szatanek R., Zembala M., Barbasz J., Czupryna A., Szczepanik A., Zembala M. (2010). Circulating tumour-derived microvesicles in plasma of gastric cancer patients. Can. Immunol. Immunother..

[26] Kim H.K., Song K.S., Park Y.S., Kang Y.H., Lee Y.J., Lee K.R., Kim H.K., Ryu K.W., Bae J.M., Kim S. (2003). Elevated levels of circulating platelet microparticles, VEGF, IL-6 and RANTES in patients with gastric cancer: Possible role of a metastasis predictor. Eur. J. Can..

[27] Rabinowits G., Gerçel-Taylor C., Day J.M., Taylor D.D., Kloecker G.H. (2009). Exosomal MicroRNA: A Diagnostic Marker for Lung Cancer. Clin. Lung Can..

[28] Chen Y.-T., Sarangadharan I., Sukesan R., Hseih C.-Y., Lee G.-Y., Chyi J.-I., Wang Y.-L. (2018). High-field modulated ion-selective field-effect-transistor (FET) sensors with sensitivity higher than the ideal Nernst sensitivity. Sci. Rep..

[29] Liu H.-H., Lin H.-Y., Liao C.-Z., Chyi J.-I. (2013). Growth and Characterization of Crack-Free Semi-Polar (1-101) GaN on 7°-off (001) Si Substrates by Metal-Organic Chemical Vapor Deposition. ECS J. Solid State Sci. Technol..

[30] Chiu H.-C., Lin C.-W., Kao H.-L., Lee G.-Y., Chyi J.-I., Chuang H.-W., Chang K.-J., Gau Y.-T. (2012). A gold-free fully copper metalized AlGaN/GaN power HEMTs on Si substrate. Microelectron. Reliab..

[31] Hsu C.-P., Chen P.-C., Pulikkathodi A.K., Hsiao Y.-H., Chen C.-C., Wang Y.-L. (2017). A Package Technology for Miniaturized Field-Effect Transistor-Based Biosensors and the Sensor Array. ECS J. Solid State Sci. Technol..

